# Mediators of Inflammation and Angiogenesis in Chronic Spontaneous Urticaria: Are They Potential Biomarkers of the Disease?

**DOI:** 10.1155/2017/4123694

**Published:** 2017-09-05

**Authors:** Ilaria Puxeddu, Federico Pratesi, Domenico Ribatti, Paola Migliorini

**Affiliations:** ^1^Clinical Immunology Unit, Department of Clinical and Experimental Medicine, University of Pisa, Pisa, Italy; ^2^Department of Basic Medical Sciences, Neurosciences and Sensory Organs, University of Bari Medical School and National Cancer Institute “Giovanni Paolo II”, Bari, Italy

## Abstract

In chronic spontaneous urticaria (CSU), different pathophysiological mechanisms, potentially responsible for the development of the disease, have been recently described. It is likely that the activation of skin mast cells with consequent release of histamine and other proinflammatory mediators is responsible for vasodilation in the lesional skin of CSU. However, the underlying causes of mast cell activation in the disease are largely unknown and remain to be identified. Thus, in this review, we discuss new insights in the pathogenesis of CSU, focusing on inflammation and angiogenesis. The understanding of these mechanisms will enable the identification of biomarkers useful for the diagnosis, follow-up, and management of CSU and will allow the development of novel, more specific, and patient-tailored therapies.

## 1. Introduction

Chronic urticaria (CU) is a common disease impacting negatively on multiple aspects of patients' lives. According to the recent guidelines, CU is defined as a disease characterized by the development of recurrent itchy wheals and/or angioedema occurring for 6 weeks or more and is divided in two major subtypes: chronic spontaneous urticaria (CSU) and inducible urticaria [1].

In the last decade, different pathophysiological mechanisms, potentially responsible for the development of CSU, have been described. It is likely that the activation of skin mast cells with consequent release of histamine and other proinflammatory mediators is responsible for vasodilation in the lesional skin of CSU [2]. However, the underlying causes of mast cell activation in the disease are largely unknown and remain to be identified. An autoimmune mechanism has been proposed, following the detection in a sizable subgroup of CSU patients of circulating anti-IgE or anti-Fc*ε*RI antibodies [3], or IgE antithyroid peroxidase (anti-TPO) [4]. This concept has led to investigate novel approaches for targeting circulating IgE in CSU [5].

However, the observation that a large part of CSU has no autoantibodies suggests that other mechanisms are probably involved in the pathogenesis of the disease and alternative therapies targeting these pathways are needed [6].

## 2. Role of Inflammatory Mediators in CSU

It is well known that mast cells are the primary effector cells in urticaria: their degranulation leads to the immediate release of preformed granular mediators such as histamine, tryptase, chymase, and proteases [2, 7]. Activated mast cells can also very rapidly synthesize and release prostaglandin- (PG-) D2, thromboxanes, leukotrienes (LTs), and platelet-activating factor (PAF). These mediators are responsible for the vasodilatation, increased vascular permeability, and stimulation of sensory nerve endings in the skin, leading to swelling, redness, and itchiness. Mast cells are also important sources of an array of cytokines, growth factors, and chemokines which may amplify and perpetuate the inflammatory state of urticaria. We have to take into account that besides an IgE-mediated mechanism, mast cells may be activated by IgG-dependent triggers or by several nonimmunological agents such as compound 48/80, basic polypeptides (polylysine, polyarginine), morphine sulphate, substance P, and the anaphylatoxin C5a [2], suggesting that mast cell activation during CSU might be the consequence of several different stimuli.

In parallel to mast cells, basophils also appear to be involved in CSU pathogenesis. In the peripheral blood of CSU patients with high disease activity, basophils are dramatically reduced, and this may be due to their recruitment from the circulation into the skin lesions [8]. Besides their reduction in the peripheral blood of severe CSU patients, basophils also present some functional abnormalities. In fact, hyporesponsiveness of these cells to anti-IgE [9] and alteration of signal transduction pathways have been reported in at least half of the patients with active disease [9–11]. For example, one of these pathways involves the histamine-releasing factor/translationally controlled tumor protein (HRF/TCTP), a cytokine which directly induces histamine release from basophils, by signal transduction process involving Syk kinase, mimicking many of the events associated with IgE-mediated activation [11].

Interesting clues to understand the mechanisms underlying CSU come from skin biopsy specimens. Besides an increased number of mast cells, a perivascular infiltrate of CD4+ lymphocytes [12], with variable numbers of monocytes, neutrophils, eosinophils, and basophils [13, 14] was demonstrated in biopsies from urticarial wheals. The cytokine profile is characterized by an increase in IL-4, IL-5, and interferon-*γ* (IFN-*γ*), which is suggestive of a mixed Th1/Th2 response [14, 15]. Cytokines that promote a Th2 profile of inflammation such as IL-33, IL-25, and thymic stromal lymphopoietin (TSLP) are increased in lesional but not in uninvolved skin, suggesting that innate pathways might play a role in the pathogenesis of CSU by activation of mast cells in the lesional skin [16]. The presence of eosinophils in biopsies from affected skin supports the view that several pathological features of CSU are in common with the allergen-induced late-phase allergic reaction [17]. Mediators released by skin mast cells following their degranulation may contribute to eosinophil recruitment, activation, and survival, leading to the perpetuation of the clinical features of urticaria. How eosinophils and their mediators may directly contribute to the development of CSU is presently unclear. However, the detection of eosinophil-derived major basic protein (MBP) in the lesional skin of CSU patients and the capacity of MBP to activate mast cells through an IgE-independent mechanism support the idea that eosinophils may directly affect mast cell degranulation with consequent amplification and perpetuation of the local inflammation in CSU [17].

Besides an increase of inflammatory mediators in the skin, several independent studies demonstrate an increase of proinflammatory cytokines in the circulation of CSU patients. IL-6, one of the main inducer of the acute-phase response of inflammation, is increased in the plasma of CSU patients and correlates with the clinical activity score of the disease. In addition, plasma IL-6 concentration is significantly lower upon spontaneous remission, suggesting that this cytokine might be a marker of disease activity [18, 19].

Among IL-1 family cytokines, the proinflammatory cytokine IL-18, initially identified as a major inducer of IFN-*γ* in Th1 and NK cells, was evaluated in the circulation of CSU and conflicting results have been reported [20–22]. Although the study of Tedeschi et al. [20] did not detect significant differences in IL-18 levels between the CSU and control group, our data indicate that in CSU both total and free IL-18 are increased [22]. Like in other inflammatory conditions characterized by high levels of IL-18, its soluble inhibitor IL-18 binding protein (BP), which regulates the activity of the cytokine, is also increased, in the attempt to counteract the proinflammatory effects of IL-18 [23].

Parallel to IL-1 family cytokines, a role of the IL-23/IL-17 axis and TNF-*α* in the pathogenesis of CSU was hypothesized [24]. High serum levels of IL-17, IL-23, and TNF-*α* were detected in CSU patients, and the levels of IL-23 and TNF-*α*, but not that of IL-17, correlated with the activity of the disease, suggesting their contribution to CSU pathogenesis and their potential role as CSU biomarkers. Recently, the role of IL-13 and periostin, involved in allergic inflammatory processes, has been investigated in CSU patients. Interestingly, while a significant increase in IL-13 was demonstrated in the CSU patients, periostin was significantly reduced in CSU patients, especially in those with severe versus mild disease [25], suggesting that the two mediators may be independently related to the pathogenesis of CSU. The inflammatory status of CSU is also supported by the results of Kaplan. In their study, blood levels of C-reactive protein (CRP), an acute-phase reactant belonging to classical short pentraxins, were found significantly higher in CSU patients compared to healthy subjects [26]. Since CRP is an acute-phase protein produced primarily in the liver under the stimulus of IL-1, TNF-*α*, and/or IL-6, it is conceivable that the increase of proinflammatory mediators and the consequent increase of CRP are hallmarks of an inflammatory condition of CSU patients [26]. Parallel to CRP, Kasperska-Zajac et al. have investigated the role of other members of the pentraxin family, in particular pentraxin 3 (PTX3), that is produced at the site of inflammation. The observation that PTX3 levels are increased in the plasma of CSU patients compared to healthy subjects may suggest a local inflammation due to activation of leukocytes that infiltrate the skin. Thus, the observed correlation between PTX3 and CRP in CSU patients suggests that these two pentraxins may be upregulated by the same mechanisms associated with acute-phase response [27].

On the basis of these findings, Bingham suggests that CSU is an immune-mediated inflammatory disease resulting from immunological activation events following exposure to exogenous or modified endogenous triggers in the presence of susceptibility factors [28]. Therefore, the inflammatory cascade may be a consequence of disturbances of innate and adaptive immunity [29], which leads to the recruitment of inflammatory and immune cells in the derma.

In this scenario, the chemokine signalling, mainly involved in the regulation of leukocyte trafficking [30], may be one of the main mechanisms responsible for the recruitment of inflammatory cells in the lesional skin of CSU. Some of the chemokines involved in CSU pathogenesis might exert their effect not only by recruiting leukocytes in the tissue but also by activating mast cells in the lesional skin. For example, CCL5/RANTES, CCL2/MCP-1, and CXCL8/IL-8 are able to induce histamine and serotonin release by mast cells, suggesting their contribution to the development of urticaria by a direct effect on mast cell degranulation [31]. Besides the role of CCL5/RANTES in the recruitment of eosinophils, monocytes, and lymphocytes in the lesional skin [32], recently it has been shown that CCL5/RANTES is able to induce the migration of progenitor mast cells and their further differentiation and activation in the tissue. The effect of this chemokine on progenitor mast cells seems to be mediated by CCR5, chemokine receptor for CCL5/RANTES also expressed on progenitor mast cells [31]. Thus, since CCL5/RANTES can be produced from mast cells in the tissue and from circulating inflammatory cells following tissue infiltration, its production and its effects in the skin of CSU can persist over time contributing to the amplification and perpetuation of the inflammatory process.

Up to now, no biomarker useful for evaluation and management of patients with CSU is available. However, recent studies assessed the relevance of laboratory markers for determining the severity or predicting the evolution of disease in adult patients with CSU [33]. Among markers of activation of the extrinsic coagulation pathway, prothrombin fragment 1 + 2, D-dimer, and CRP are increased in CSU patients and seem to correlate with disease severity. In particular, the D-dimer level significantly correlates with UAS in CSU as well as in acute urticaria, suggesting its role as a marker of disease severity in both forms of urticaria [34]. Parallel to the coagulation/fibrinolysis pathways, an imbalance in pro- and anti-inflammatory adipokines in CSU patients has been also observed. In particular, according to the results of Trinh et al., lipocalin-2 (LCN2) might be used as a marker not only of disease activity but also of the clinical response to antihistamine treatment [35], suggesting new approaches to monitor disease progression and response to therapy in CSU patients. Potential disease markers have also been investigated in the presence of other comorbidities. Recently, in Korean patients with CU and metabolic syndrome (MS), a hospital-based cross-sectional study demonstrated a correlation between uncontrolled CU and the levels of C3, TNF-*α* and ECP. However, it is unclear whether the increased systemic inflammation is just an epiphenomenon or has any role in the pathogenesis of CU associated with MS [36].

## 3. Differences in ASST-Positive and ASST-Negative Subgroups of CSU

ASST is widely used in the diagnosis of CU in order to evaluate an autoimmune origin of the disease. In fact, a correlation between a positive ASST and the presence of anti-Fc*ε*RI and anti-IgE antibodies was reported. On the contrary, it is still debated whether or not this test has any prognostic value. Some studies demonstrated an increased disease activity or a longer duration of urticaria in ASST-positive versus ASST-negative patients, while others did not report significant differences between the two subgroups in terms of severity, disease duration, and quality of life scores [37–40]. Interestingly, in a study conducted by Ye et al. [41], it has been shown that ASST reactivity was a significant predictor of well-controlled CU during the 6-month stepwise treatment according to the recent guidelines. Since the reactivity of ASST is mainly due to serum factors responsible for histamine release and vasodilatation that are controlled by antihistamines, the authors hypothesized that ASST-positive patients are expected to achieve a well-controlled state within 6 months of treatment. Therefore, according to these data, the results of the ASST may be a useful parameter for predicting response to treatment and monitoring therapeutic response in patients with CU.

## 4. Role of Endothelium and Coagulation System in CSU

Besides the chemokine system, the endothelium plays a critical role in controlling the passage of fluid into the tissue and influencing cellular trafficking [42]. In the skin, endothelium dysfunction might increase vascular permeability, with a consequent proinflammatory response. The soluble forms of adhesion molecules, such as vascular cellular adhesion molecule-1 (VCAM-1) and intercellular adhesion molecule (ICAM-1), are widely used as biomarkers of endothelial dysfunction, and their increase in the circulation and skin biopsies [32] seems to reflect a proinflammatory endothelium phenotype in several skin diseases, including CSU [14, 32, 43, 44].

Recent studies on coagulation performed in CSU patients have produced interesting results [45]. According to these studies, the coagulation cascade seems to be activated in CSU, involving the extrinsic pathway first and the intrinsic pathway secondarily [45–47]. The detection of increased levels of factor VIIa, prothrombin fragment 1 + 2, and D-dimer in CSU suggests that, following endothelial cell activation, tissue factors are released with consequent activation of the extrinsic coagulation cascade and secondary fibrinolysis [46, 47]. Thus, these results are of particular interest when considering that thrombin can increase vascular permeability and is a potent inducer of mast cell degranulation, at least in experimental models [45]. Furthermore, the plasma levels of D-dimer, frequently elevated in patients with severe CSU, seem to decrease following treatment with omalizumab [48], suggesting a link between circulating autoantibodies, activation of coagulation, and fibrin degradation in severe CSU.

## 5. Role of Mediators Regulating Angiogenesis in CSU

Angiogenesis is the growth of new blood vessels from preexisting ones. It is a multistep and highly orchestrated process involving vessel sprouting, endothelial cell migration, proliferation, tube formation, and survival [49]. Under physiologic conditions, angiogenesis depends on the balance of positive and negative angiogenic mediators within the vascular microenvironment and requires the functional activities of a number of molecules, including angiogenic factors, extracellular matrix proteins, adhesion receptors, and proteolytic enzymes [49]. Angiogenesis is also associated with pathologic conditions as direct response to tissue demands, such as chronic inflammation, fibrosis, and tumor growth [50].

Numerous inducers of angiogenesis have been identified, including members of the fibroblast growth factor (FGF) family, vascular permeability factor/vascular endothelial growth factor (VEGF), angiogenin, transforming growth factor alpha and beta (TGF-*α* and TGF-*β*), platelet-derived growth factor (PDGF), TNF-*α*, hepatocyte growth factor/scatter factor (HGF/SC), granulocyte macrophage colony-stimulating factor (GM-CSF), and angiopoietin-1 and angiopoietin-2.

Among them, VEGF is the most potent direct-acting regulator of angiogenesis, and its expression is often excessive in chronic inflammatory diseases. VEGF induces proliferation, migration, and tube formation of endothelial cells. It promotes secretion of interstitial matrix metalloproteinase-1 (MMP-1) and von Willebrand factor and the expression of chemokines, as well as leukocyte adhesion molecules, such as ICAM-1, VCAM-1, and E-selectin [51]. VEGF is also a potent survival factor for endothelial cells, and it induces in endothelial cells the expression of antiapoptotic proteins. VEGF also causes vasodilatation through the induction of the endothelial nitric oxide (NO) synthase and the subsequent increase in NO production. Therefore, VEGF acts principally on endothelial cells, even though it can influence other cell types, including hematopoietic stem cells, monocytes, and other inflammatory cells.

Recently, the presence of new blood vessels in the skin of CSU patients has been reported by Kay et al. [17]. They demonstrated that the lesional skin of CSU patients contained significantly more CD31-positive endothelial cells compared to the normal skin. Increased vascularity was also confirmed by confocal imaging using the lectin *Ulex europaeus* agglutinin 1 (UEA-1). In the same skin lesions, the increase of new vessels parallels the increased numbers of eosinophils, neutrophils, basophils, and macrophages, suggesting a direct contribution of these inflammatory cells to the formation of blood vessels. This is the first report showing angiogenesis in the skin lesions of CSU, but previous data were consistent with this observation. In fact, increased levels of VEGF have been observed in the circulation and in the tissue of CSU patients [52]. Thus, we can suggest that in CSU VEGF induces vascular leakage as well as the formation of new vessels. Since VEGF is mainly produced by inflammatory cells, we can also hypothesize that mast cells and infiltrating eosinophils and basophils present in skin lesions might contribute to the release of VEGF with consequent increase in vascular permeability and neoangiogenesis. On the other side, mast cells, eosinophils, and basophils might be a target for VEGF, leading to perpetuation and amplification of the inflammatory processes [53]. The functional activity of VEGF is tightly regulated by endogenous antiangiogenic mediators mainly produced by the degradation of extracellular matrix (ECM) components such as endostatin (ES) and thrombospondin- (TSP-) 1. Recently, we have reported increased levels of ES and TSP-1 in the sera of CSU patients, which do not correlate with the activity of the disease [54]. Thus, these antiangiogenic mediators, able to exert multiple activities during inflammation and angiogenesis, might be involved in the pathogenesis of CSU. We have to take into account that besides their antiangiogenic activities, ES and TSP-1 also exert other important roles in the skin remodelling. For example, TSP-1 destabilizes a contact between endothelial cells due to its direct effect on the cells, contributing to skin vasodilation and consequent extravasation [55] and ES, a proteolytic fragment of collagen type XVIII, acts as a vasoactive mediator due to its direct effect on endothelial cells via NO synthesis [56]. Therefore, both ES and TSP-1 might contribute to the vascular leakage in CSU, leading to the development of its clinical manifestations, such as wheals and flare formation.

Parallel to ECM fragments, some members of MMPs are increased in the circulation of CSU patients [57]. For example, MMP-9, an endopeptidase involved in ECM degradation during inflammation, tissue remodelling, and angiogenesis, is increased in the peripheral blood of CSU patients in adults and in children [58]. Some [57–59], but not all studies [60], reported an association between disease activity and plasma concentration of MMP-9. We can hypothesize that together with VEGF and ECM fragments, the increase of MMP-9 in CSU might contribute to both vascular leakage and angiogenesis, leading to the amplification and perpetuation of the inflammatory process. It is likely that MMP-9 contributes to the pathogenesis of CSU as well as that of other chronic diseases (i.e., asthma) where inflammation and tissue remodelling take place.

## 6. Conclusion and Prospective

In CSU, several processes such as inflammation, coagulation, and angiogenesis take place. We have discussed new insights that demonstrate the active contribution of inflammatory cells (mast cells, basophils, eosinophils, neutrophils, and lymphocytes), cytokines, growth factors, soluble adhesion molecules, ECM fragments, and MMPs in the development of CSU. Many of the abovementioned cells and mediators seem to be involved in the pathogenesis of the disease, but none of these seems to be really specific to CSU. Furthermore, some of them correlate with the urticaria activity score, but so far are not used as disease biomarkers. The understanding of the mechanisms underlying the pathogenesis of CSU will enable the identification of biomarkers useful for the diagnosis, follow-up, and management of the disease and will allow the development of novel, more specific, and patient-tailored therapies.
